# One Dimensional AuAg Nanostructures as Anodic Catalysts in the Ethylene Glycol Oxidation

**DOI:** 10.3390/nano10040719

**Published:** 2020-04-10

**Authors:** Daniel K. Kehoe, Luis Romeral, Ross Lundy, Michael A. Morris, Michael G. Lyons, Yurii K. Gun’ko

**Affiliations:** School of Chemistry, Trinity College Dublin, Dublin 2, Ireland; kehoeda@tcd.ie (D.K.K.); ROMERALA@tcd.ie (L.R.); LUNDYRO@tcd.ie (R.L.); MORRISM2@tcd.ie (M.A.M.); MELYONS@tcd.ie (M.G.L.)

**Keywords:** ultrathin, nanowire, anodic catalyst, tunable

## Abstract

Direct alcohol fuel cells are highly promising as efficient power sources for various mobile and portable applications. However, for the further advancement of fuel cell technology it is necessary to develop new, cost-effective Pt-free electrocatalysts that could provide efficient alcohol oxidation and also resist cross-over poisoning. Here, we report new electrocatalytic materials for ethylene glycol oxidation, which are based on AuAg linear nanostructures. We demonstrate a low temperature tunable synthesis that enables the preparation of one dimensional (1D) AuAg nanostructures ranging from nanowires to a new nano-necklace-like structure. Using a two-step method, we showed that, by aging the initial reaction mixture at various temperatures, we produced ultrathin AuAg nanowires with a diameter of 9.2 ± 2 and 3.8 ± 1.6 nm, respectively. These nanowires exhibited a high catalytic performance for the electro-oxidation of ethylene glycol with remarkable poisoning resistance. These results highlight the benefit of 1D metal alloy-based nanocatalysts for fuel cell applications and are expected to make an important contribution to the further development of fuel cell technology.

## 1. Introduction

Over the last few decades, alcohols such as methanol and ethanol have been extensively studied as highly promising fuels for fuel cells [1,2,3]. While these alcohols provide high energy densities, they have significant disadvantages; methanol has a low boiling point and is toxic, while ethanol, which is produced from biomass, requires a vast amount of land and infrastructure. In addition, both of these fuels have a high tendency to cross over the polyelectrolyte membrane and poison the cathode, thus hampering the performance of the fuel cells [4,5,6,7]. In more recent years, researchers have moved towards the use of ethylene glycol (EG) as a fuel [8,9]. EG offers a higher energy density and, due to its size, is less likely to cause crossover poisoning. More importantly, EG is already produced on a large scale for the car industry (such as for coolant antifreeze), thus the potential resources are already present. Unlike methanol, which is one of the simplest fuels, having no C–C bonds, the oxidation of EG is more complex. It is generally accepted that the oxidation of EG in an alkaline medium occurs via a poisoning or non-poisoning pathway [10,11,12]. In the non-poisoning route, EG is completely oxidised to oxalate, while in the poisoning route EG forms formate as an intermediate, which subsequently oxidises to form poisoning species.

While Pt- and Pd-based nanomaterials have commonly been used as fuel cell catalysts, there is a major effort to move to more cost-efficient materials. Researchers have focused on alloying as an amenable solution to producing more sophisticated catalysts that are not only cheaper, but also offer improved atom utilisation and catalytic properties [13,14,15,16]. In particular, Au- and/or Ag-containing alloys have emerged as effective anodic catalysts for fuel cell applications, most notably for the EG oxidation reaction (EGOR) [11,17,18,19]. Due to advances in synthetic methods, these noble metal nanomaterials can be produced in a broad range of shapes and sizes, making it possible to manipulate catalyst design [20,21,22,23,24]. 

Furthermore, the use of 1D Pt and Pd nanomaterials, alloyed or decorated with Au, has shown significant catalytic activity owing to their synergistic effects and large surface areas [18,25,26,27,28,29,30,31]. To this end, ultrathin nanowires (NWs) have become widely used in fuel cells, offering remarkable catalytic performances [32,33,34,35,36,37]. Typically, templating methods relying on galvanic replacement are a popular route to producing and controlling the size of 1D ultrathin NWs [38,39,40]. The drawback of templating, however, is that it requires additional synthetic steps, materials of suitable redox potential and can often result in some of the templating metal(s) being retained in the final structure. While there are many examples of the ligand-controlled synthesis of Au- and Ag-based ultrathin NWs, there still remains a lack of tunable wet chemical synthesis methods. One such example by Xu et al. [29] demonstrated a facile synthesis of ultrathin PdAu NW networks of different sizes by varying the solvent used in the reaction. Thus, if we could develop more tunable wet chemical methods that do not involve templating and are free of Pt and/or Pd, it would facilitate the design of new, more efficient and sustainable electrocatalysts. Nanostructures based on AuAg alloys in particular have not only very strong plasmonic characteristics, but also possess interesting electrocatalytic properties [41]. This was recently highlighted by Xu et al. [42], who showed that the white light irradiation of AuAg nanobowls results in amplifying their performance for EGOR. This has also been shown for a variety of related materials, such as AuAg nanoparticles [43], PtAg dendrites [44] and PtAu@Pt dendrites [45], with each material offering a pronounced improvement in performance when irradiated with white light. Thus, the need to develop anodic catalyst-containing plasmonic metals is a necessary step to producing new, smart and cost-effective fuel cell technology.

While the synthesis and application of ultrathin Au-based NWs has been previously reported [46,47,48,49,50], their use as anodic catalysts is still in its infancy [51]. Here, for the first time, we report an optimisation of a tunable synthesis of 1D AuAg ultrathin nanostructures and explore the use of ultrathin AuAg NWs as anodic catalysts for the electro-oxidation of EG.

## 2. Materials and Methods 

### 2.1. Materials 

HAuCl_4_·3H_2_O (Sigma-Aldrich, Arklow, Ireland, ≥ 99.9), AgNO_3_ (Sigma-Aldrich, Arklow, Ireland, 99.99%), polyvinylpyrrolidone (PVP) (Sigma-Aldrich, Arklow, Ireland, average M_w_ 40,000), L-ascorbic acid (Sigma-Aldrich, Arklow, Ireland, 99%), N,N-dimethylformamide (DMF) (ACS regent, ≥ 99.8%), ethylene glycol (Sigma-Aldrich, Arklow, Ireland, 99%) and KOH (Fischer scientific, Dublin, Ireland). Millipore water was obtained using a Milli-Q filtration system operating at 18 ΩM.

### 2.2. Characterisation Techniques 

TEM was performed on a JEOL 2100 electron microscope. HRTEM, STEM and EDX were performed on an FEI Titan transmission electron microscope (FEI-ThermoFischer Scientific, Eindhoven, Netherlands). UV-Vis absorption spectroscopy was performed on a LAMBDA 1050 UV/vis/NIR spectrometer (ThermoFischer Scientific, Eindhoven, Netherlands) using a quartz cuvette with a 1 cm path length. XRD crystallography measurements were performed on a Bruker D2 Phaser diffractometer (Bremen, Germany). X-ray photoelectron spectroscopy (XPS, VG Scientific ESCAlab Mk II) was performed under ultra-high vacuum conditions (< 5 × 10^−10^ mbar) using a hemispherical analyser and Al Kα X-rays (1486.6 eV). The emitted photoelectrons were collected at a take-off angle of 90° from the sample’s surface. The analyser pass energy was set to 100 eV for survey scans and 30 eV for high-resolution core scans, yielding an overall resolution of 1.2 eV. Photoemission peak positions were corrected to C 1s at a binding energy of 284.8 eV. Peak-fitting analysis was performed using CasaXPS (Ver. 2.3.19). The Au 4f and Ag 3d spectra were fitted using a Shirley background and a Voigt function. Curve fitting was carried out for features with marked asymmetry indicative of overlapping peak contributions. Peaks were unconstrained, the goodness of fit was manifested in residuals, the peak area ratio of the individual contribution was as expected for spin-orbit contributions and peak widths were consistent with the literature.

### 2.3. Synthesis and Characterisation of 1D AuAg Nanostructures

PVP (1 mL, DMF, 500 mM), HAuCl_4_·3H_2_O (200 µL, DMF, 50 mM) and AgNO_3_ (200 µL, aqueous, 50 mM) were dissolved in DMF (8 mL). The solution was vortexed for 2 min and an aqueous solution of ascorbic acid (1 mL, 400 mM) was then injected into the solution. The mixture was vortexed (using an IKA Genius 2 vortex mixer) for a further 30 s and then left standing for 18 h at 20 and 25 °C to produce AuAg NWs with average diameters of 9.2 ± 2 and 3.8 ± 1.6 nm, respectively. Aging the reaction mixture at 35 and 40 °C resulted in AuAg nano-necklace structures and nanodisks, respectively. After the aging step, the solution was then diluted by a factor of 20 with water to produce the final structure. The resulting dark grey solution was then centrifuged twice (9000 rpm, 35 min) and the precipitate was re-dispersed in water.

### 2.4. Synthesis and Ultrathin AuAg Nanowires as Anodic Catalysts for Ethylene Glycol Oxidation

The glassy carbon working electrode (3 mm diameter) was modified with 9.2 ± 2 or 3.8 ± 1.6 nm ultrathin AuAg NWs by drop casting from stock solutions and allowed to dry in air. Typically, 10 µg of catalyst in each case was used. Nafion (25 µL) 1 wt % was then drop cast onto the modified electrode and allowed to air dry. The cyclic voltamograms (CVs) were obtained in nitrogen-saturated solutions and the potential was typically scanned from −0.8 to 0.8 V (versus a saturated camel electrode reference electrode) at 50 mV·s^−1^. All measurements were carried out using a solution of 0.5 M ethylene glycol with 1 M KOH as the electrolyte. The scan was repeated several times to ensure that a stable and reproducible CV curve was obtained.

## 3. Results and Discussion

### 3.1. Synthesis and Characterisation of 1D AuAg Nanostructures

**Figure 1:**
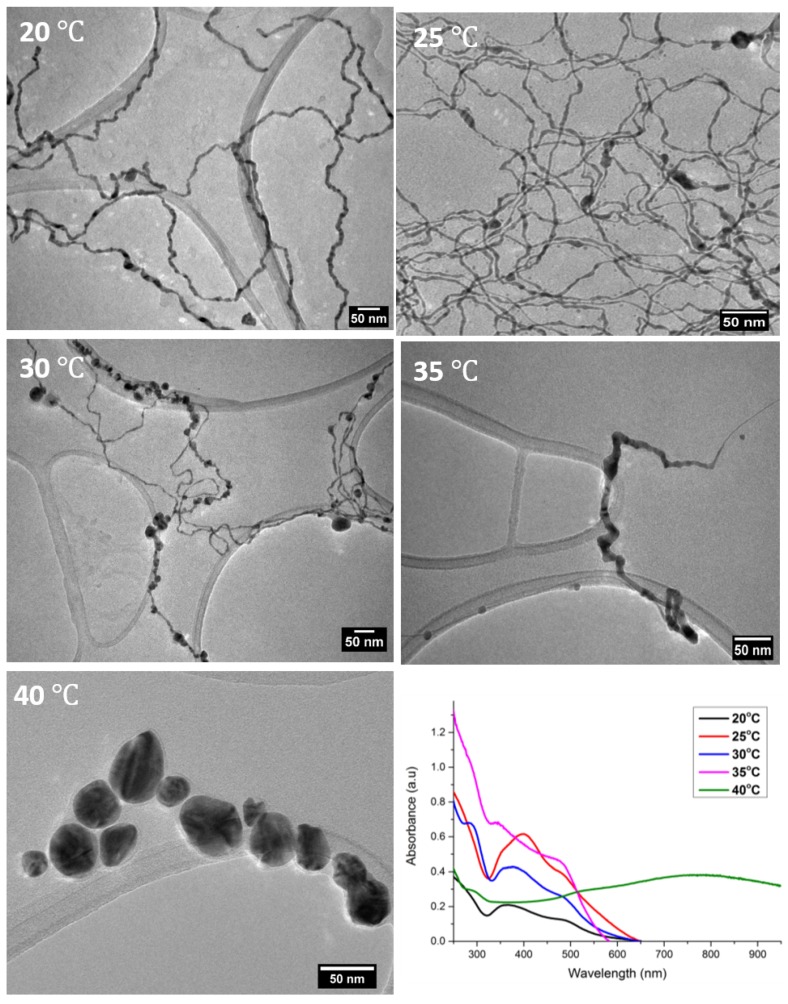
TEM and UV-Vis analysis of products following dilution after aging at various temperatures for 18 h.

Previously, we have reported the synthesis of ultrathin AuAg nanowires by the reaction of HAuCl_4_·3H_2_O and AgNO_3_ with ascorbic acid in the presence of PVP. Here, we present new synthetic approaches which enabled us to produce various types of 1D AuAg nanostructures with minimal polydispersity. The new synthetic process involved the preparation and controlled aging of the initial seeding reaction mixture at different temperatures (20, 25, 30, 35 and 40 °C) for 18 h following the addition of ascorbic acid. The solutions were then diluted by a factor of 20 with water and the products were investigated by TEM (Figure 1). 

**Figure 2:**
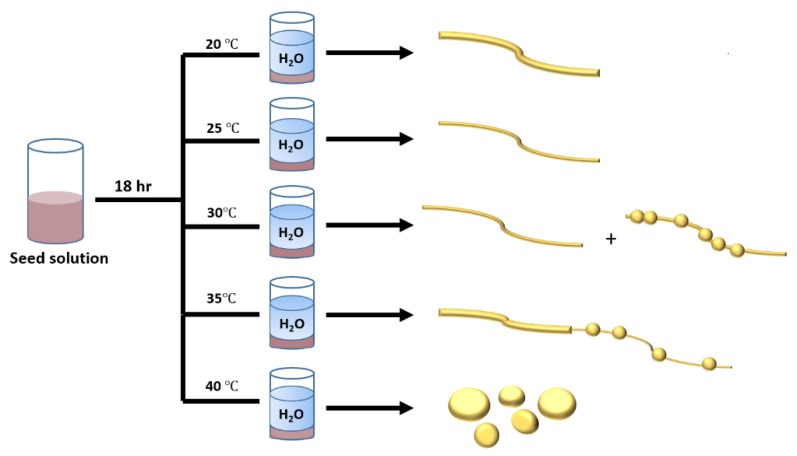
Schematic summarising the synthesis and products following dilution after aging at various temperatures for 18 h.

The TEM images showed a significant difference in the resulting nanostructures’ size and morphology between various temperature regimes. Aging at 20 °C followed by dilution resulted in non-uniform NWs with an average diameter of 9.2 ± 2 nm (see Figures S1 and S2 in Electronic Supporting Information (ESI) for additional TEM images and size distributions, respectively). Interestingly, after aging at 25 °C, the average diameter of the post-dilution NWs was reduced to 3.8 ± 1.6 nm (refer to Figure S3 in ESI for size distribution), and they were also highly monodispersed. This difference in product after a small temperature change of 5 °C highlights the sensitivity of this process to temperature. It was noted that when the reaction mixture was aged at 30 °C, the products became polydisperse. We observed that two types of nanomaterials emerged: one which was similar in size and morphology to the 25 °C-aged sample, while the other type appeared more necklace-like, containing bead-like shapes along the length of the NW (Figure S4, ESI). After aging the sample at 35 °C, a complete morphology change occurred, with the presence of only ultrathin nano-necklace-like structures. A detailed study of these necklace structures will be reported separately. Thus, aging at 30 °C marked a transition point in the morphology of the resulting NWs. Finally, aging at 40 °C proved to be critical as only large anisotropic disk-shaped nanoparticles of various shapes and sizes were formed (refer to Figure S5 for additional TEM images of products). The UV-vis analysis (Figure 1) of all the products from this study showed peaks at 500 and 370 nm in all cases except for the 40 °C aged sample, which displayed peaks at 525 and 780 nm, which is common for disk-like nanoparticles [52,53,54]. The peaks noted at 370 and 500 nm have been previously reported for ultrathin AuAg NWs [46,55] and most likely correspond to the longitudinal and transverse modes of the structures due to the non-uniformity along their length [56,57]. All solutions had a dark black-grey colour, except the solution of the product obtained at 40 °C (the disks), which had a dark navy colour. A schematic presentation of the synthesis and product nanostructures is shown in Figure 2 below.

We propose that the 1D nanomaterials were formed via a two-step process involving the orientated attachment of Au and Ag seeds during the aging period to form ultrathin NWs [58]. Following the dilution step, the excess seeds in dispersion underwent a controlled fusion with the ultrathin NWs to form the resulting thicker NWs [59,60]. Therefore, keeping the reaction mixture at a fixed temperature served to accelerate the nucleation and growth processes of the template NWs during aging, resulting in fewer excess seeds in solution [61]. This accounts for the size difference between the NWs formed after aging at 20 and 25 °C. It is clear that, as the temperature was elevated, it played a more significant role. In the case of the highest studied temperature (40 °C), Oswald ripening completely dominated the growth process, resulting in only nanoparticles [62,63], while at 30 and 35 °C, we saw that the final structures were composed of both NW and nanoparticle features. Given that the lower temperatures favoured nanowires and the highest temperature produced nanoparticles, it is expected that this morphology change occurred. In addition, the polydispersity of the products was notably reduced, as the growth kinetics were not altered due to temperature fluctuations during the aging period. 

**Figure 3:**
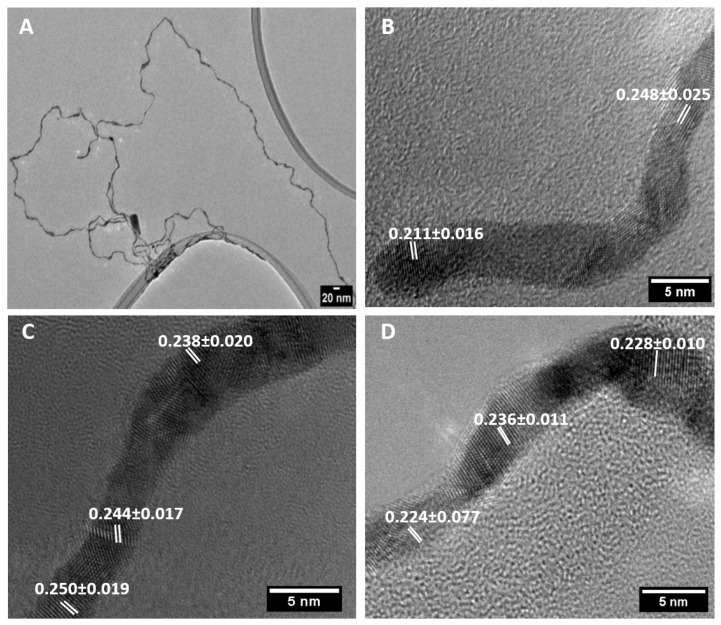
TEM image (**A**) and HRTEM images (**B**–**D**) of ultrathin AuAg nanowires (NWs) produced following dilution after aging at 25 °C for 18 h. All d-spacing values are expressed in nanometres.

We conducted a further detailed characterisation of 1D AuAg nanostructures produced following dilution after aging at 25 °C. The HR-TEM analysis (Figure 3) showed that these nanomaterials were polycrystalline and exhibited multiple lattice fringes with d values of 0.236 ± 0.011 and 0.244 ± 0.025 nm, corresponding to the (111) and (200) of FCC Au and Ag.

**Figure 4:**
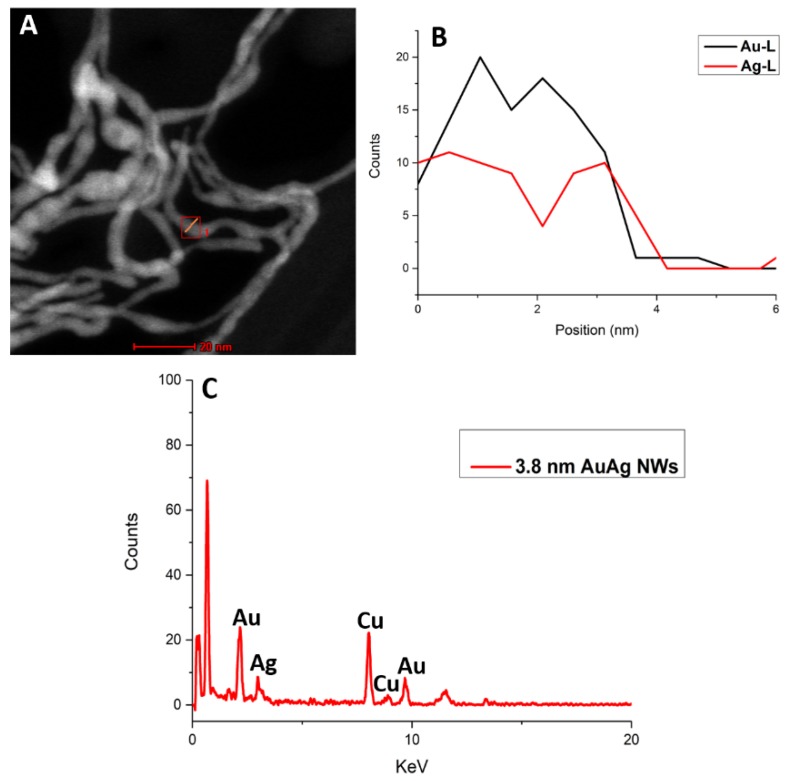
High-angle annular dark-field scanning transmission electron microscopy (HAADF-STEM) image highlighting region of interest (**A**), EDX line profile (**B**) and corresponding EDX spectrum (**C**) of AuAg NWs produced following dilution after aging at 25 °C.

The EDX line map analysis further confirmed that the 1D nanomaterials were an alloy of Au and Ag with an Au:Ag ratio of 70:30 (Figure 4). The EDX spectrum shows the characteristic L and M peaks of Au at 9.7 and 2.1 KeV, respectively, and the L peak of Ag at 3.1 KeV. The additional peaks were due to the Cu from the TEM grids used for this analysis (refer to Figure S6 for HAADF-STEM image of NWs produced after aging at 20 °C). 

**Figure 5:**
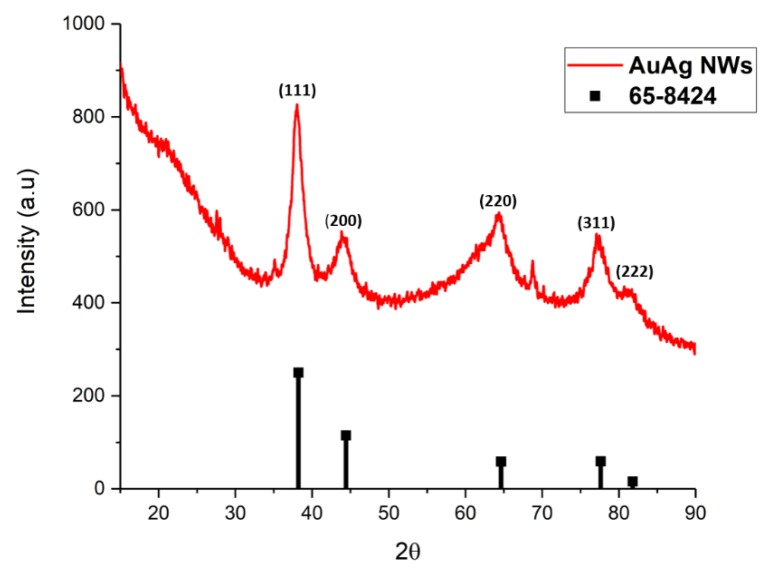
XRD pattern of AuAg NWs produced following dilution after aging at 25 °C.

The XRD analysis (Figure 5) showed the expected pattern with diffraction peaks at 37.8°, 44.3°, 64.3°, 77° and 82°, corresponding to the (111), (200), (220), (311) and (222) planes, respectively, for FCC AuAg (65-8424).

**Figure 6:**
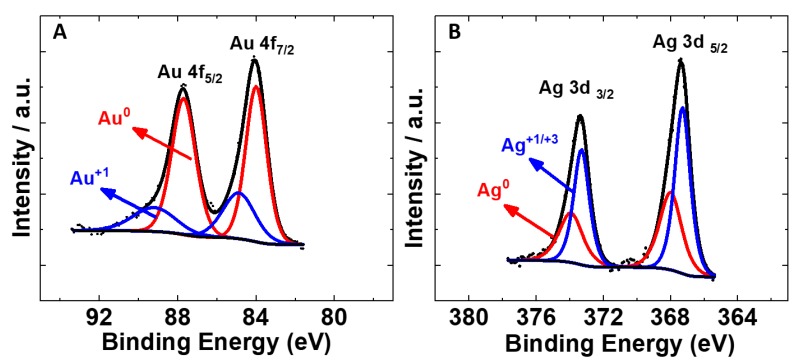
XPS spectra of Au (**A**) and Ag (**B**) components of nanowires produced following dilution after aging at 25 °C for 18 h.

High-resolution XPS spectra for Au and Ag (Figure 6) are shown for the 25 °C sample [64,65,66]. The Au high-resolution scan in Figure 6A has peaks at 83.9 and 87.6 eV, corresponding to the 4f_7/2_ and 4f_5/2_ of Au^0^ [67]. The curve fitting revealed that the higher binding energy contributions shifted in binding energy by ~1.5 eV, indicative of the presence of some Au^+1^ species [68]. For Ag (Figure 6B), the high-resolution spectra indicate the presence of Ag^0^ peaks at 368.1 and 374.1 eV for 3d_5/2_ and 3d_3/2_. The peak shifts of ~0.7 eV at lower binding energies show the presence of Ag^+1^/Ag^+3^ species [69]. We note that, due to the very close proximity (~0.3 eV), we were unable to distinguish between the Ag^+1^/Ag^+3^ oxidation states. See supporting information (Figure S7) for additional XPS data for other samples. The different relative metal:oxide peak area contributions were due to different mean free electron paths of metal signals through a surface oxide layer. The ratio of oxide:metal signals was within expectations of the known passivation layer thickness of these metals [70,71].

### 3.2. Electro-oxidation of Ethylene Glycol Using Selected AuAg 1D Nanostructures as Anodic Catalysts

**Figure 7:**
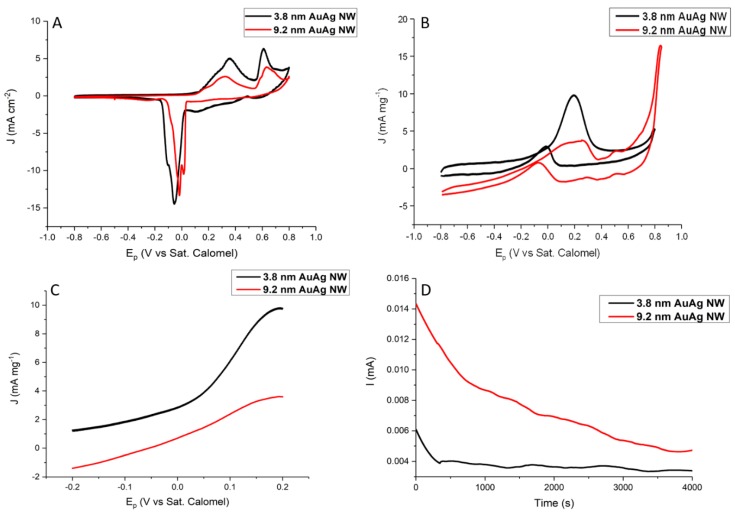
Cyclic voltamogram (CV) curves (**A**) of 3.8 and 9.2 nm AuAg NWs in N_2_-saturated 1M KOH at 50 mV·s^−1^. (**B**) CV curves, (**C**) enlarged CV curves in the forward scan and (**D**) *I*–*T* curves over 4000 s for 3.8 and 9.2 nm AuAg NWs in N_2_-saturated 1 M KOH solution containing 0.5 M ethylene glycol (EG) versus the sat. calomel reference electrode.

The potential electro-catalytic activities of the 9.2 ± 2 and 3.8 ± 1.6 nm diameter AuAg NWs were assessed by cyclic voltammetry (CV) in a 1 M KOH solution containing 0.5 M EG (Figure 7). For each electrochemical test, a 10 µg loading of catalyst was drop cast onto the glassy carbon electrode (GCE). CV curves (after several scans) from each NW in a N_2_-saturated solution of 1M KOH at a scan rate 50 mV·s^−1^ are shown in Figure 7A. The peaks at 0.38 V in both cases in the anodic sweep were due to the formation of Au oxides and Ag_2_O layers, while the peak at 0.6 V was associated with the oxidation of Ag_2_O to AgO. In the cathodic sweep, the small peak noted at 0.1 V was due to the reduction of Ag_2_O to Ag. The peaks at 0.02 and 0V in the case of the 9.2 nm NWs, and 0.05 and 0.1V for the 3.8 nm NWs, were due to the reduction in Au oxides and the desorption of hydroxide ions, respectively. The current was normalised to the geometric area of the GCE. The electro-oxidation of EG is a complex process and can involve the formation of various partially oxidised products. Figure 7B shows that in the anodic sweep both NWs produced a large peak due to the oxidation of EG. It was noted that the 3.8 nm NWs produced a larger mass activity (9.6 mA·mg^−1^) compared to the thicker NWs (4.1 mA·mg^−1^) and that the peak potential of these thinner NWs was slightly lower (0.193 V) than the thicker NWs, which had a peak potential at 0.22 V. These results highlight the influence of the NW diameter on catalytic performance, with thinner NWs benefiting from larger surface areas and better surface atom utilisation, offering more active sites compared to the thicker NWs. It is well recognised that oxide species, such as adsorbed hydroxides, play a key role in the electro-oxidation of alcohols. Thus, as the potential in the CV is increased (towards 0.8 V), the AuAg surface of the NWs becomes oxidised, which blocks OH from adsorbing to active sites [72,73]. In the cathodic sweep, this oxide layer is reduced, facilitating the adsorption of hydroxides and the subsequent oxidation of chemi-adsorbed surface poison species at 0.1 V in the case of both NWs. Furthermore, the larger the ratio of the forward peak (*J*_f_) to the reverse peak (*J*_b_), the greater the electrode’s poison-resistance ability [74,75,76]. In our case, *J*_f_/*J*_b_ for the 3.8 ± 1.6 and 9.2 ± 2 nm NWs was found to be 3.2 and 1.7, respectively. While the PVP layer has been reported to affect the accessibility of alcohols to the catalyst’s surface, it has been widely used on a range of electro-catalysts and does not significantly inhibit their overall performance [77,78,79,80]. Compared with other catalysts (Table 1), our NWs, particularly the 3.8 nm diameter NWs, have a remarkable catalytic performance with a poison resistance comparable to many of the state-of-the-art noble metal catalysts. Mechanistically, it is generally accepted that for AuAg alloys, the electro-oxidation of alcohols occurs on Au sites [81,82]. The role of Ag in these alloys is to promote oxygenated species on the catalyst surface and has been shown to alter the electronic properties of Au, resulting in weaker binding between the Au active sites and the alcohol [13,83,84,85]. Using chronoamperometric *I*–*T* curves over a 4000 s period (Figure 7D) at 0.2 V, it was further found that the thicker NWs were more durable than the thinner NWs, as noted by the 12% higher current output compared to the thinner NWs after the studied time (please see Supporting Information for calculation). It is well established that as NWs get smaller in diameter, they become poorer electrical conductors. Thus, we propose that, in our case, the thinner NWs had less metallic behaviour and were consequently not as conductive and durable as the thicker NWs under the applied potential.

## 4. Conclusions

In conclusion, we have demonstrated that maintaining a fixed temperature during the aging period of the initial AuAg nanowire mixtures resulted in a range of new 1D nanomaterials with low polydispersity and enabled us to tune the size and morphology of the resulting products. Importantly, both of the ultrathin AuAg NWs studied in this work showed remarkable electrocatalytic activity as anodic catalysts for the EGOR. It was noted that the thinner NWs produced higher current densities at a lower potential compared to the larger NWs, as a result of their larger surface area. We believe that this tunable synthesis and the ultrathin 1D AuAg nanostructures produced will pave the way for developing advanced Pt-free anodic catalysts and will open new horizons in the use of AuAg-based alloys for future fuel cell applications.

## Figures and Tables

**Table 1:** Electro-oxidation parameters of EG by various noble metal-based catalysts in an alkaline medium.

Catalyst	*E*_p_ (V)	*J*_f_/*J*_b_
Au nanostars [24]	0.2 (vs. sat. calomel)	3.71
AuPd@Pd nanocrystals [86]	ca. −0.7 (vs. sat. calomel)	2.41
Au nanocrystals [86]	ca. 0.3 (vs. sat. calomel)	2.31
AuPd NW networks [29]	ca. 0.025 (vs. sat. calomel)	ca. 0.8
AuPd Nanoflowers [79]	ca. −0.01 (vs. sat. calomel)	ca. 0.75
Hollow AuAg_1_Cu_1_ nanoflowers [87]	−0.22 (vs. sat. calomel)	ca. 2.6
3D Au_33_Cu_67_ ultrathin NW network [51]	ca. 0.3 (vs. sat. calomel)	ca. 4.2
PdAuRu nanocrystals [88]	−0.1 (vs. sat. calomel)	ca. 1.92
Open bowl-like Pt_1_Au_1_Ag_1_ nanocages [19]	ca. 0.6 (vs. sat. calomel)	ca. 4.7
3D Pt_5.7_Pb ultrathin NW networks [89]	ca. −0.1 (vs. sat. calomel)	ca. 5.7
Pt_31_Cu_69_ NWs [90]	−0.1 (vs. sat. calomel)	ca. 1.97
Screw-like PtPd NWs [91]	ca. 0.1 (vs. sat. calomel)	ca. 0.842
Pt_1_Co_1_ NWs [92]	ca. 0.505 (vs. sat. calomel)	ca. 1.26
AuAg NWs (9.2 nm) (this work)	0.22 (vs. sat. calomel)	1.7
AuAg NWs (3.8 nm) (this work)	0.193 (vs. sat. calomel)	3.2

## Supplementary Materials

The following are available online at https://www.mdpi.com/2079-4991/10/4/719/s1, Figure S1: TEM images of nanowires produced following dilution after aging at 20 °C for 18 h; Figure S2: Size distribution of ultrathin AuAg NWs produced following dilution after aging at 20 °C for 18 h with average diameter of 9.2 ± 2 nm; Figure S3: Size distribution of ultrathin AuAg NWs produced following dilution after aging at 25 °C for 18 h with average diameter of 3.8 ± 1.6 nm; Figure S4: TEM images of nanowires produced following dilution after aging at 30 °C for 18 h; Figure S5: TEM images of nanoparticles produced following dilution after aging at 40 °C for 18 h; Figure S6: STEM image of nanowire produced following dilution after aging at 20 °C for 18 h; Figure S7: XPS analysis of nanowires produced following dilution after aging at 20 °C for 18 h, Durability calculation of anodic catalysts.
