# Supplementary material for: One Dimensional AuAg Nanostructures as Anodic Catalysts in the Ethylene Glycol Oxidation
Source: Nanomaterials (Basel). 2020 Apr 10;10(4):719. doi: 10.3390/nano10040719 (PMC7221585; doi:10.3390/nano10040719)
Supplement: Supplementary file 1 [file nanomaterials-10-00719-s001.pdf]

Supplementary Materials

One Dimensional AuAg Nanostructures as Anodic Catalysts in the Ethylene Glycol Oxidation

Daniel K. Kehoe, Luis Romeral, Ross Lundy, Michael A. Morris, Michael G. Lyons and Yurii K. Gun'ko *

School of Chemistry, Trinity College Dublin, Dublin, Dublin 2, Ireland; kehoeda@tcd.ie (D.K.); ROMERALA@tcd.ie (L.R.); LUNDYRO@tcd.ie (R.L.); MORRISM2@tcd.ie (M.M.); MELYONS@tcd.ie (M.L.)

* Correspondence: IGOUNKO@tcd.ie; Tel.: +353-1-896-3543

Received: 28 February 2020; Accepted: 6 April 2020; Published: date

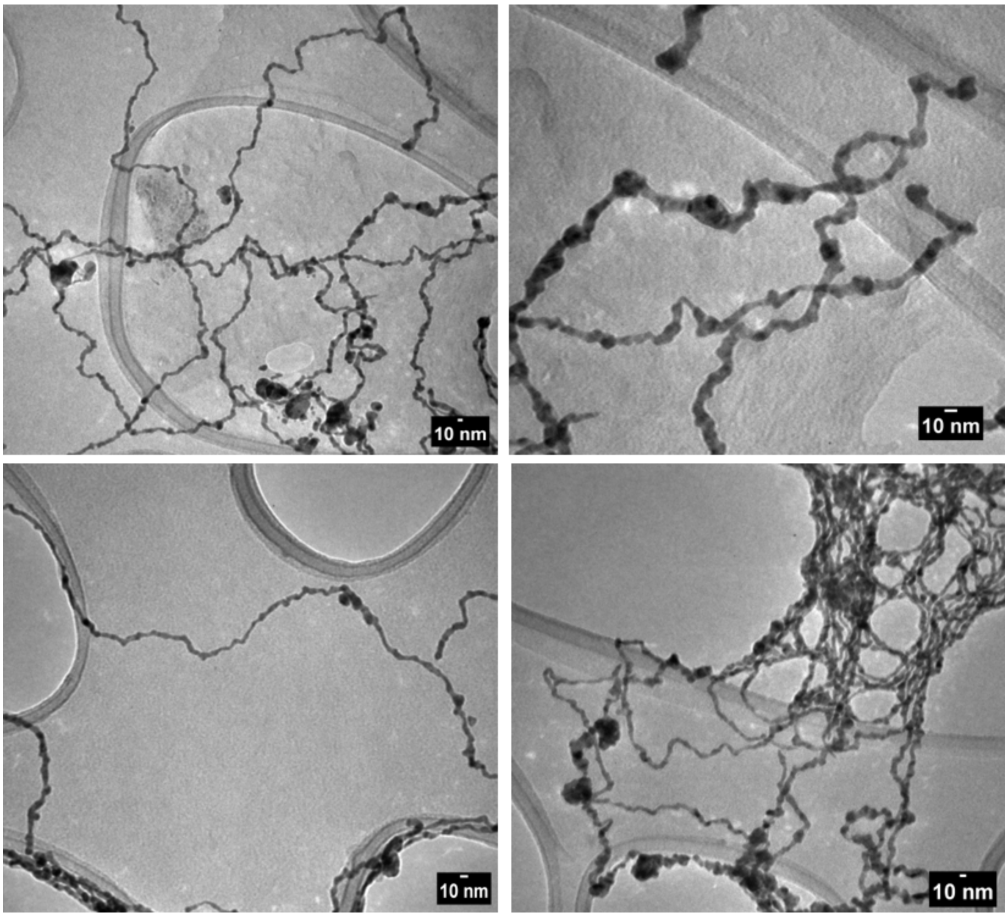

Figure S1: TEM images of nanowires produced following dilution after aging at 20 °C for 18 h.

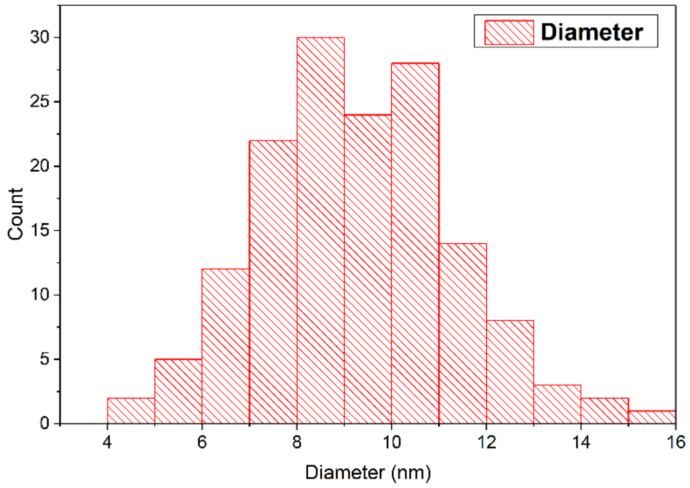

Figure S2: Size distribution of ultrathin AuAg NWs produced following dilution after aging at 20 °C for 18 h with average diameter of 9.2 ± 2 nm.

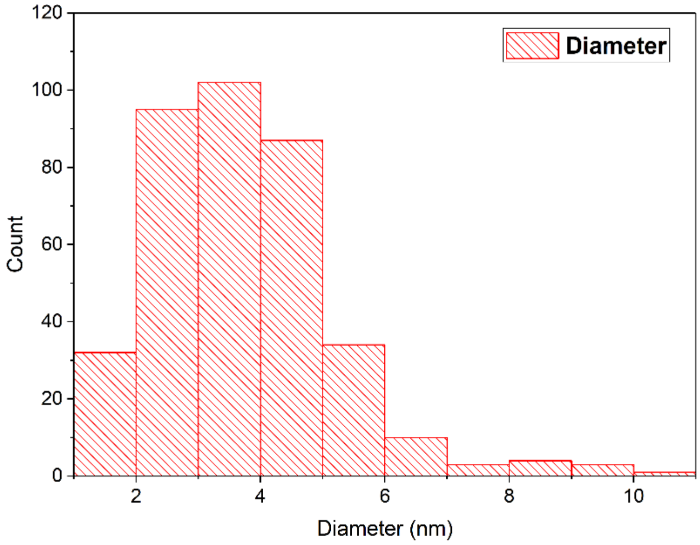

Figure S3: Size distribution of ultrathin AuAg NWs produced following dilution after aging at 25 °C for 18 h with average diameter of 3.8 ± 1.6 nm.

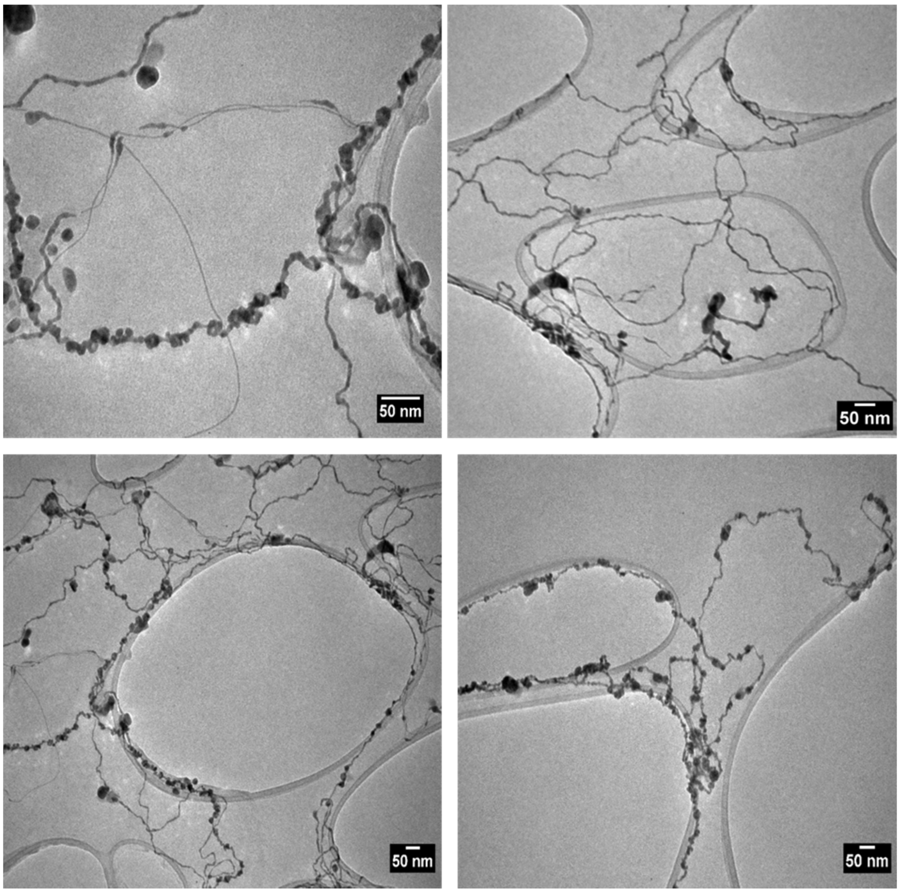

Figure S4: TEM images of nanowires produced following dilution after aging at 30 °C for 18 h.

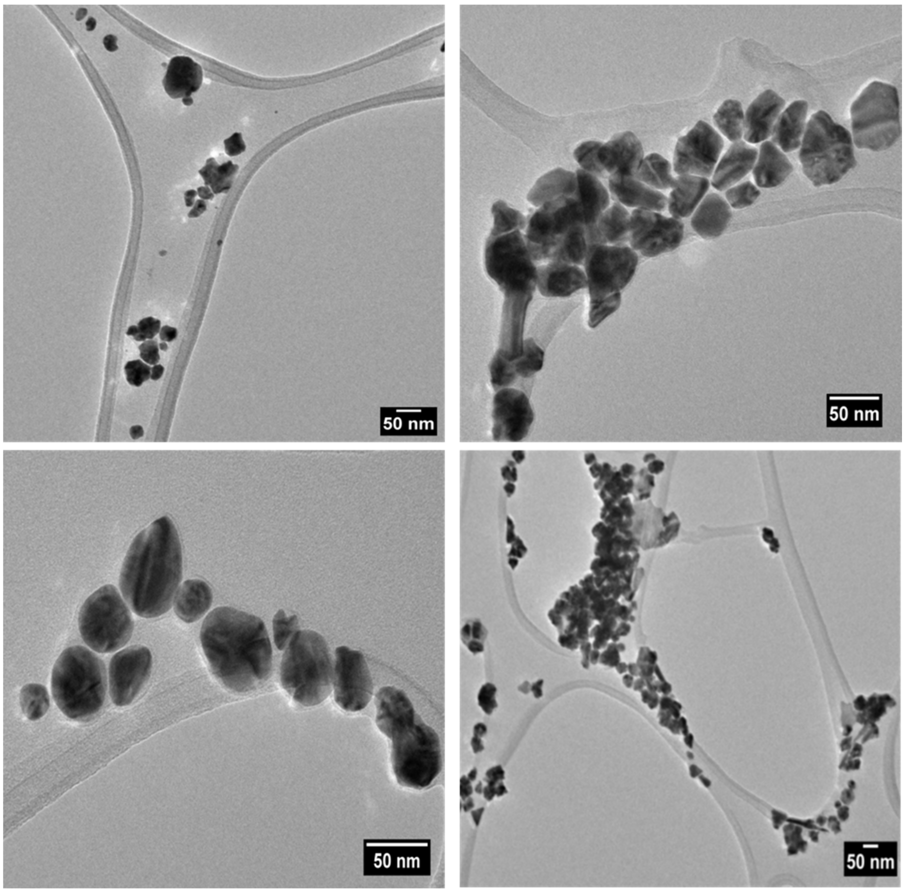

Figure S5: TEM images of nanoparticles produced following dilution after aging at 40 °C for 18 h.

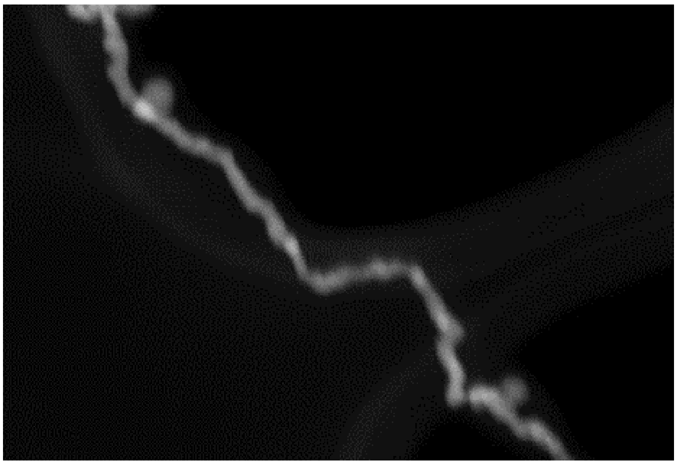

Figure S6: STEM image of nanowires produced following dilution after aging at 20 °C for 18 h.

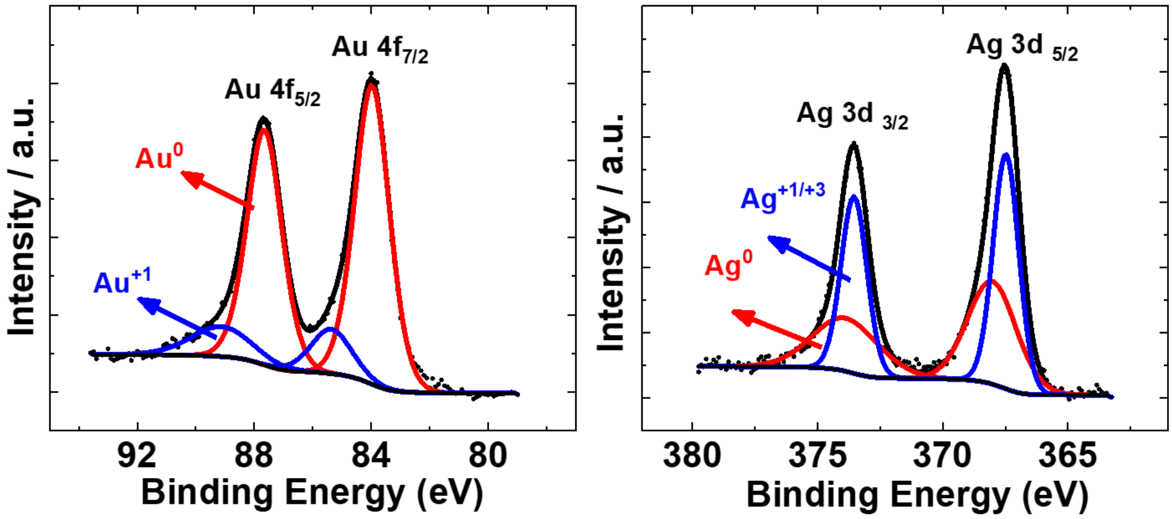

Figure S7: XPS analysis of nanowires produced following dilution after aging at 20 °C for 18 h.

Durability calculation of AuAg NWs from chrono-amperimetric *I*–*T* curves

$$\frac{\boldsymbol{i_t}}{\boldsymbol{i_0}} \times \mathbf{100\%}$$

9.2 nm AuAg NWs

$$\frac{0.0047}{0.0142} \times 100\% = 33\%$$

3.8 nm AuAg NWs

$$\frac{0.0033}{0.006} \times 100\% = 55\%$$
